# High‐intensity interval training attenuates cardiac injury by targeting ferroptosis and endoplasmic reticulum‐stress in male rats with heart failure

**DOI:** 10.14814/phy2.70580

**Published:** 2025-09-25

**Authors:** Aida Sabouri, Abbasali Gaeini, Soheila Adeli, Alireza Ghardashi Afousi

**Affiliations:** ^1^ Electrophysiology Research Center, Neuroscience Institute Tehran University of Medical Science Tehran Iran; ^2^ Department of Exercise Physiology, Faculty of Sport Science and Health University of Tehran Tehran Iran

**Keywords:** cardiac cell death, endoplasmic reticulum stress, exercise training, heart failure

## Abstract

This study investigated the effects of high‐intensity interval training (HIIT) on endoplasmic reticulum (ER) stress, ferroptosis, and iron deposition in rats with heart failure (HF). HF was induced by intraperitoneal injection of isoprenaline (130 mg/kg/day) for 4 days. Afterward, rats were divided into control healthy (Control), HF sedentary (HF‐Sed), and HF HIIT (HF‐HIT) groups. The HF‐HIT group underwent HIIT (5 intervals of 4 min at 85%–90% VO_2max_, separated by 2 min at 50%–60% VO_2max_) for 8 weeks. Biomarkers of ER stress, ferroptosis, and oxidative stress, along with cardiac function, were measured post‐intervention. HIIT reduced cardiac fibrosis and iron deposition while increasing cystine/glutamate transporter (SLC7A11), glutathione peroxidase 4 (GPX4), and SOD levels. Additionally, protein levels of glucose‐regulated protein 78 (GRP78), protein kinase RNA‐activated‐like ER kinase (PERK), and activating transcription factor 4 (ATF4) decreased after HIIT. These findings suggest that HIIT alleviates ferroptosis and ER stress via the PERK/ATF4/SLC7A11/GPX4 pathway, offering protective effects against HF.

## 1 INTRODUCTION

Heart failure (HF) is a clinical syndrome that depressed cardiac output by various structural and functional abnormalities of the heart and contributes to high morbidity and mortality worldwide (Joseph et al., 2017). Cardiomyocyte death has been identified to play an important pathophysiological role in the development of HF (Mishra et al., 2019). Iron‐dependent, non‐apoptotic regulatory cell death was recently identified, known as ferroptosis (Dixon et al., 2012). Ferroptosis is characterized by iron accumulation and lipid peroxidation (Li, Cao, et al., 2020). This process has been implicated in the pathogenesis of HF, particularly in models exhibiting reduced ejection fraction (HFrEF). The excessive iron accumulation in HF triggers ferroptotic cell death in cardiomyocytes (Wang et al., 2023; Yang et al., 2022; Zhang et al., 2023). The dysregulation of intracellular iron metabolism drives ferroptosis through Fenton reaction–mediated overproduction of reactive oxygen species (ROS) and accumulation of lipid hydroperoxides, specifically hydroperoxyphospholipids, which severely compromise cardiomyocyte viability. Accumulation of lipid peroxide resulting from lethal ROS‐Fenton reaction may inhibit the activity of cystine‐glutamate antiporter (Xc^−^or SLC7A11), which leads to decreased glutathione peroxidase 4 (GPX4) activity (Zeng et al., 2019; Wang et al., 2020; Du et al., 2021). ROS overproduction is considered a key mediator to initiate ferroptosis through transcription factor nuclear factor erythroid 2‐related factor 2 (NRF2)‐transcriptional activator of the oxidative stress response‐/GPX4 signaling pathways (Sun et al., 2016).

Previous studies have shown that endoplasmic reticulum stress (ERS) has a key role in ferroptosis through activation of the unfolding protein response (UPR) (Lee et al., 2018; Li, Li, et al., 2020). The endoplasmic reticulum is a multifunctional organelle responsible for the synthesis, folding, assembly, transport of proteins, calcium homeostasis, and phospholipid biosynthesis (Minamino et al., 2010). Disruption in ER functions, referred to as ERS, is affected by many factors such as ischemia, hypoxia, Ca^+2^ dysregulation, and oxidative stress, which lead to an accumulation of unfolded and misfolded proteins (Hetz & Papa, 2018). UPR is a compensatory pro‐survival response that induces signal transduction events to restore ER homeostasis (Minamino et al., 2010). The canonical UPR is detected by three ER transmembrane sensors, including protein kinase RNA‐activated‐like ER kinase (PERK), activating transcription factor 6 (ATF6), and inositol‐requiring protein‐1 (IRE1), which bind through the interaction with the ER‐resident chaperone glucose‐regulated protein 78 (GRP78) (Hetz & Papa, 2018). Ferroptosis could activate the PERK‐ATF4‐ and C/EBP homologous protein (CHOP) pathways, resulting in ROS overproduction induced by the interaction between iron ions and NADPH oxidase in mitochondria (Li, Li, et al., 2020).

Exercise training is an effective non‐pharmaceutical therapeutic strategy for cardiovascular disease (CVD). The evidence suggests that aerobic exercise may reduce hospitalization and improve life quality in CVD by improving cardiorespiratory fitness and cardiac function (Luo et al., 2017). A recent study indicated aerobic exercise may inhibit ferroptosis through activation of the SLC7A11/NRF2/GPX4 pathway in cerebral ischemia–reperfusion injury (Liu et al., 2022). It has been shown that upregulation of thioredoxin 1 by aerobic exercise training attenuates ERS and cardiomyocyte apoptosis following myocardial infarction (Cai et al., 2020). The growing body of evidence from human and experimental studies demonstrates that exercise intensity is a key contributor to cardio protection and the physiological response of the adult heart (Angadi et al., 2015; Waring et al., 2014). High‐intensity interval training (HIIT) has been recommended as a time‐efficient and superior alternative to traditional aerobic training in improving cardiorespiratory fitness and left ventricular function in CVD (Cai et al., 2016; Claes et al., 2017; Naderi et al., 2019). However, the effects of HIIT on ferroptosis and ERS following HF remain unclear. We hypothesized that HIIT may improve cardiac function by focusing on ferroptosis and ERS in rats with HF. This study examined the effects of HIIT on heart function, ferroptosis markers, antioxidants, and ERS pathways in rats with HF.

## 2 METHODS AND MATERIALS

### 2.1 Animals and experimental design

**FIGURE 1:**
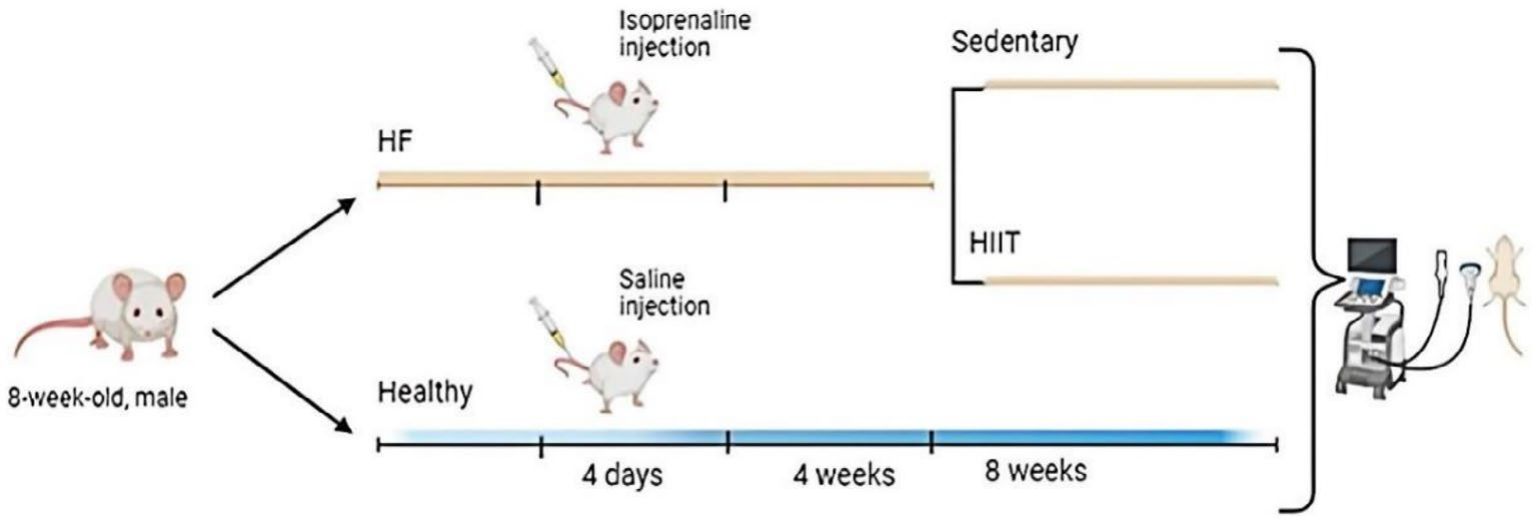
Study design overview. Eight‐week‐old male Wistar rats' littermates were in familiarization laboratory condition. Afterwards, HF‐ rats were subcutaneously injected with 1 dose of isoprenaline (130 mg/kg body weight) per day for 4 consecutive days. After another 4 weeks, HF rats were randomly divided into high‐intensity interval (HIT) and sedentary (Sed) groups. The HIT consisted of 8 weeks of treadmill running (5 intervals of 4 min at 85%–90% VO_2max_ separated by 2 min at 50%–60% VO_2max_, 3 d/wk).

Twenty‐four male Wistar rats (8 weeks old, weighing 200–250 g) used in this study were purchased from Pasteur Institute (Tehran, Iran). All rats were maintained in a specific condition (temperature: 22 ± 2°C, humidity: 60 ± 5%) with a 12 h: 12 h light/dark cycle, supplied with the standard rodent diet (Behparvar Co., Iran; Batch No. BN‐0428, Tehran, Iran) and freely available drinking water. All experimental procedures and protocols were approved by the Animal Ethics Committee of Tehran University of Medical Science (IR.TUMS.AEC.1402.097) with the Guide for the Care and Use of Laboratory Animals published by the United States National Institutes of Health (NIH Publication, 8th Edition, 2011). The animals were randomly allocated into the following three groups: (1) control health (Control, *n = 8*), (2) HF sedentary (HF‐Sed, *n = 8*), and (3) HF HIIT (HF‐HIT, *n = 8*). HF was induced by isoprenaline (Sigma Aldrich, St. Louis, MO, USA; Cat. No. 51‐30‐9, P Code: 101308221) dissolved in sterile saline and subcutaneously injected once a day (130 mg/kg/day for 4 days consecutively) (Pan et al., 2022; Riehle & Bauersachs, 2019), while the control group received sterile saline. The study design overview is depicted in Figure 1.

### 2.2 Echocardiography

The rats were anesthetized with sodium thiopental (Sigma Aldrich, St. Louis, MO, USA; Cat. No. T1022) at a dose of 25 mg/kg administered intraperitoneally. A 10‐MHz linear array transducer was used to conduct transthoracic echocardiography. The transducer was connected to a Vivid 7 expert ultrasound system (General Electric Vingmed Ultrasound, Horten, Norway). The ultrasound was performed at a speed of 100 mm/s, 4 weeks after isoprenaline injection, and at the end of the experiments for all groups. The assessment of left ventricular dimensions, including diastolic and systolic diameter internal diameter as well as posterior wall thickness in systolic and diastolic phases, was conducted using parasternal two‐dimensional (2D) and M‐mode short‐axis images at the level of the papillary muscle. The functional parameters, including fractional shortening (FS) and left ventricular ejection fraction (LVEF), were calculated using the abovementioned primary measurements (Scheer et al., 2012). All echocardiographic evaluations were performed by a cardiologist who specialized in small animal echocardiography and was blind to the groups being studied.

### 2.3 Training intervention

The rats in the HF groups underwent echocardiographic examinations and were then placed on a motorized treadmill (Iranian Danesh Salar, Tehran, Iran). They walked at a slow pace of 5 m/min for 5 min per day, 3 days per week, in order to become acquainted with the training. Subsequently, the measurement of maximal oxygen uptake (VO_2max_) was conducted during maximal exercise using the methodology outlined in a previous study (Høydal et al., 2007). The animals in the CTR and HF‐Sed groups were only positioned on the treadmill without any form of exercise training. The HIT‐HF performed 8 weeks of HIIT which every exercise session consisted of five intervals of 4 min, with an intensity of 85%–90% VO_2max_. These intervals were followed by 2 min of active recovery at 50%–60% VO_2max_. Before and after every session, the animals underwent a 5‐min warm‐up and cool‐down period at 50%–60% VO_2max_. A prior study examining the correlation between running speed and VO_2max_ determined the exercise training intensity for each week. During an 8‐week training period, the running speed was steadily increased by 0.02 m/s per week after each week of exercise training (Naderi et al., 2019). The treadmill had a 0‐degree inclination for both training and testing.

### 2.4 LDH and cTn‐I measurement

Serum levels of lactate dehydrogenases (LDH) (BXC0242, Biorexfars, Shiraz, Iran) and cardiac troponin‐I (cTn‐I) (CSB‐E06594r, Houston, TX 77054, USA) were measured by ELISA, according to the manufacturer's instructions. Intra‐ and inter‐assay coefficients of variation were <3.3 and <3.4, respectively, for LDH <8% and <10%, respectively, for cTn‐I.

### 2.5 Iron content

Tissue iron content was measured using the Ferrozine Chromogen method (Zhang et al., 2012). In brief, the tissues were homogenized and centrifuged at 6000 rpm for 15 min at 4°C, and the supernatant was collected. Subsequently, TCA solution 3% was added to each supernatant and then vortexed for 1 min. After that, the supernatant was incubated at 4–8°C for 30 min. The supernatant was then collected, and 8 μL of Ferrozine Chromogen (Sigma Aldrich, St. Louis, MO, USA; Cat. No. P9762) was added to each well, mixed, and incubated at room temperature for 5 min. The supernatant's absorbance was read at 560 nm to determine the ferrous ion levels. The results are presented as μg of iron per g of wet tissue.

### 2.6 SOD, TAC, and MDA measurements

The spectrophotometric analysis of superoxide dismutase (SOD) and malondialdehyde (MDA) was conducted using cardiac tissue homogenates, according to the manufacturer's instructions. SOD was measured by inhibiting superoxide‐induced reduced nicotinamide adenine dinucleotide (NADH) oxidation based on the Paoletti et al. method (Boitard et al., 2014) with the Ransod, Randox kit (United Kingdom). Total antioxidant capacity (TAC) was measured quantitative sandwich according to the assay kit instructions (MBS1600693, San Diego, CA 92195‐3308, USA). The levels of malondialdehyde (MDA) were determined using the thiobarbituric acid reactive substances (TBARS) method (TBARS assay kit, Teb Pazhouhan Razi, Tehran, Iran; Cat. No. TPR‐MDA‐96T) (Pozzi et al., 2013).

### 2.7 Transferrin ELISA measurement

Blood samples were collected from the inferior cava vein with EDTA solution and rapidly centrifuged (3000 rpm for 20 min at 4°C) to collect serum. Transferrin serum levels (MBS024755, MyBioSource, San Diego, USA) were measured by ELISA, according to the manufacturer's instructions. Intra‐ and inter‐assay coefficients of variation were less than 15%.

### 2.8 Western blot of protein expression

Western blot was performed as described in a previous study (Naderi et al., 2019). Heart tissue was homogenized with RIPA buffer containing protease inhibitors (Sigma‐Aldrich, Cat. No. 20‐188, CA, USA) and then centrifuged at 14,000 rpm at 4°C for 20 min. The Bradford method was used to determine protein concentration according to the manufacturer's instructions (Sigma‐Aldrich, Cat. No. B6916, 82024 Taufkirchen, Germany). Equal amounts of protein (20 μg) were mixed with Laemmli sample buffer (2X) supplemented with β‐mercaptoethanol and boiled at 95°C for 5 min. Next, proteins were separated by SDS‐polyacrylamide gel electrophoresis (10%–12%) and transferred to polyvinylidene difluoride (PVDF) membranes (Bio‐Rad Laboratories, Cat. No. 162‐0177, CA, USA). The PVDF membranes were blocked overnight with 5% BSA (Sigma Aldrich, Cat. No. A‐7888, MO, USA) with 0.1% Tween 20 at room temperature. Membranes were then incubated overnight at 4°C with the following primary antibodies in TBS‐Tween 20%–5% BSA: rabbit polyclonal anti‐FPN1 (5 μg/mL; Abcam, Berlin, Germany; Cat. No. ab235166), anti‐SLC7A11 (1:1000; Abcam, Berlin, Germany; Cat. No. ab307601), anti‐GPX4 (1:5000; Abcam, Berlin, Germany; Cat. No. ab125066), anti‐NRF2 (1:1500; Abcam, Berlin, Germany; Cat. No. ab313825), anti‐GRP78 (1 μg/mL; Abcam, Berlin, Germany; Cat. No. ab21685), anti‐ATF6 (1 μg/mL; Abcam, Berlin, Germany; Cat. No. ab37149), anti‐CHOP (1:5000; Abcam, Berlin, Germany; Cat. No. ab233121), anti‐β‐actin (1:2500; Abcam, Berlin, Germany; Cat. No. ab8227), phospho Ser40‐NRF2 (1 mg/mL; Biorbyt, North Carolina, USA; Cat. No. orb6544), anti‐PERK (1:1000; Cell Signaling Technology, Leiden, Netherlands; Cat. No. 11815S), anti‐ATF4 (1:1000; Cell Signaling Technology, Leiden, Netherlands; Cat. No. 11815S), and anti‐ERO1 (1:1000; Biomol GmbH, Hamburg, Germany; Cat. No. E3452‐85) at room temperature. Subsequently, membranes were washed with TBST buffer three times (15 min) and then incubated with secondary antibody: goat anti‐rabbit IgG HRP (1:10000; Abcam, Berlin, Germany; Cat. No. ab6721) for 2 h at room temperature. The membranes were then incubated with enhanced chemiluminescence (ECL) reagent (Thermo Fisher Scientific, Waltham, USA; Cat. No. 32106) for 1–2 min. Densitometry of protein bands was quantified by gel analyzer Version 2010a software (NIH, USA), such that the percentage area under the curve of each band was divided by the percentage area under the curve of its corresponding actin band, and then calculated values were compared between groups and expressed as a ratio of the loading control. The proteins were expressed to β‐actin, and phosphorylated proteins were expressed relative to total protein. The same β‐actin blot is shown in all figure panels where protein expression is reported. This is because all target proteins were probed using the same gel and membrane, and the same β‐actin blot served as the loading control for all experiments. This has been clearly stated in the figure legends.

### 2.9 Quantitative real‐time PCR

TRIzol reagent (Invitrogen, Carlsbad, CA, USA; Cat. No. 15596026) was used to extract total RNA from the cardiac tissue. The RNA concentration and purity were then measured using a spectrophotometer. The cDNAs were generated using the Transcript First‐Strand cDNA Synthesis Super Mix (TransGen Biotech, Beijing, China; Cat. No. AT301‐02) following the instructions provided by the manufacturer. The Rotor‐Gene 6000 system (Corbett, Concorde, NSW, Australia) utilized TransStart Top Green qPCR SuperMix (TransGen Biotech, Beijing, China; Cat. No. AQ131‐01) for real‐time PCR. The PCR‐reaction mixes consisted of 5 μL of TransStart Top Green qPCR SuperMix, 1 μL of template cDNA, 1 μL of forward and reverse primers, and 3 μL of sterile distilled water. The thermal protocol that was suggested involved an initial denaturation step at a temperature of 95°C for a duration of 2 min. This was followed by 40 cycles of denaturation at 95°C for 10 s, followed by an extension step at 60°C for 30 s. In order to mitigate RNA degradation, all procedures were conducted under cold conditions. The housekeeping gene employed in this study was β‐actin, and the relative expression analysis was conducted using the 2^−ΔΔCt^ technique. The reactions were performed in triplicate, and the investigators were blinded to the group assignments during all the analyses. Table 1 presents the forward and reverse primer sequences (Naderi et al., 2019). All primers were synthesized by Macrogen Inc., Seoul, South Korea.

**TABLE 1:** primer sequences for RT‐qPCR.

Gene	Forward (5′‐3′)	Reverse (5′‐3′)
TRF‐R1	ACTTCTTCCGTGCTACTTCCAG	ACTCCACTCTCATGACACGATC
FTL	CAGCCTGGTCAATTTGTACCT	GCCAATTCGCGGAAGAAGTG
FTH	AAGCTGCAGAACCAACGAGG	AGTCACACAAATGGGGGTCATT
β‐actin	CGGTCAGGTCATCACTATCGG	ATGCCACAGGATTCCATACCCA

Abbreviations: FTH, ferritin heavy chain; FTL, Ferritin light chain; TRF‐R1, Transferrin receptor1.

### 2.10 Cardiac fibrosis

Whole hearts were harvested, fixed in 4% paraformaldehyde, and embedded in paraffin, and serially cut into 6‐μm thick sections from base to apex. The sections were stained with Masson's Trichrome. Images were captured using a Nikon Eclipse E200 microscope (Nikon Corporation, Tokyo, Japan) equipped with a 40× objective lens. For each rat, three adjacent sections were quantified using ImageJ software (version 1.53, NIH, Bethesda, MD, USA).

### 2.11 Statistical analysis

Data were expressed as mean ± standard deviation (SD) and analyzed via the Statistical Package for Social Science (SPSS, version 25.0; SPSS Inc., Chicago, IL, USA). GraphPad Prism was used to create the graphs (Version 9, Boston, MA 02110, USA). The level of statistical significance was set at *p* < 0.05. A Shapiro–Wilk test was used to confirm that the distribution of all outcome variables was normal. One‐way ANOVA followed by Tukey's post hoc test was used to determine differences between groups.

## 3 RESULTS

### 3.1 Effects of HIIT on body weight, heart weight, and heart weight/body weight ratio after HF

There are no significant differences in the body weight, heart weight, and heart weight/body weight ratio following long‐term HIIT in all groups (Table 2). Then, HIIT would not impact the overall health situation of HF rats.

**TABLE 2:** Body and heart weights, and heart weight/body weight ratio after experimental exercise training.

	Control	HF‐sed	HF‐HIT
BW (g)	336.8 ± 22.3	346.1 ± 32.8	324.5 ± 30.2
HW (mg)	1023.3 ± 145.7	1083.3 ± 25.1	1180 ± 301.3
HW/BW ratio (mg/g)	2.9 ± 0.2	3.2 ± 0.3	3.7 ± 1.1

*Note*: Values are presented as means ± SD.

Abbreviations: BW; body weight, HW; heart weight.

### 3.2 HIIT improves cardiac function in HF

**FIGURE 2:**
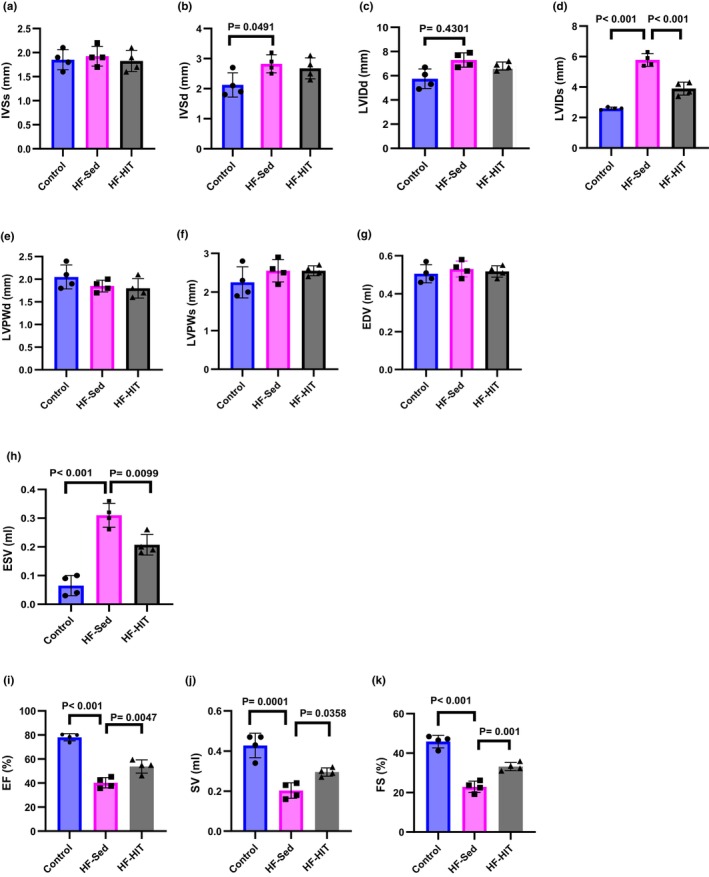
(a–k) Cardiac function was examined by echocardiography (*n* = 8). EDV, end‐diastolic volume; ESV, end‐systolic volume; EF, ejection fraction; FS, fractional shortening; IVSd; interventricular septal end diastole; IVSs, interventricular septal end systole; LVIDd, LV diameter at end‐diastolic phase; LVIDs, LV diameter at end‐systolic phase; LVPWd, posterior wall thickness at end‐diastolic phase; LVPWs, posterior wall thickness at end‐systolic phase; SV, stroke volume. Data are presented as means ± SD; dots represent individual rats. Exact *p* values are shown on the graph.

Our results confirm that HIIT improves myocardial contractility by increased EF and FS in HF‐HIT versus HF‐Sed (*p* = 0.001). Additionally, it is demonstrated that IVSd (*p* = 0.049) increased in HF‐Sed versus control, but this increase is not seen in IVSs. Eight weeks of HIIT had no influence on IVSd, IVSd, and LVIDd in HF‐HIT, while LVIDs decreased in HF‐HIT versus HF‐Sed (*p* = 0.001). The results show that HIIT increased SV values (*p* = 0.049) in HF‐HIT versus HF‐Sed were accompanied by a decrease of ESV (*p* = 0.010). (Figure 2a–k).

### 3.3 HIIT improves running distance

**FIGURE 3:**
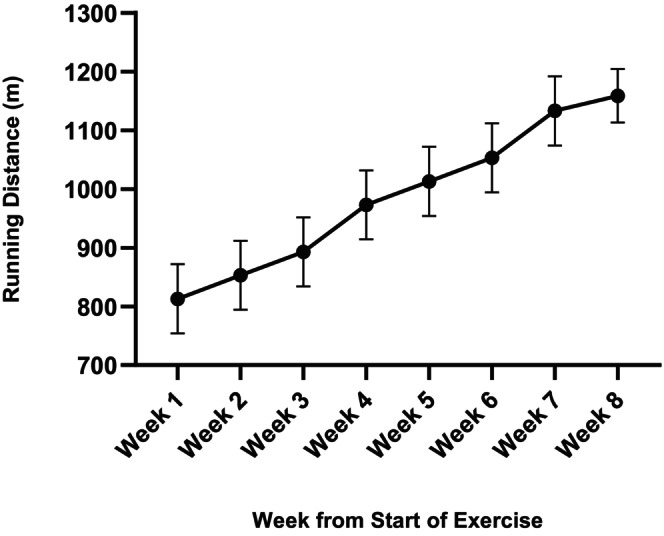
Weekly running distance progression in rats on a treadmill.

The running distance of HF‐HIT rats on the treadmill has a progressive increase from the first week to the last week. The distance of running of HF‐HIT increased by 44.3% from the last week versus the first week. All the rats ran 812 m in the baseline and progressively increased to 1172 m in the final week. The mean running speed across all HF‐HIT rats was 20.32 m/min in Week 1 and increased to 28.96 m/min in Week 8, reflecting the progressive overload of the training protocol (Figure 3). Therefore, HIIT would improve cardiorespiratory fitness in HF rats.

### 3.4 HIIT ameliorates cardiac injury index

**FIGURE 4:**
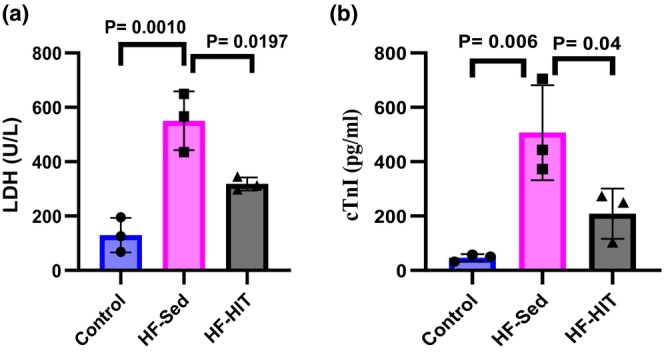
(a, b) Serum levels of LDH, cTnI (*n* = 8). Data are presented as means ± SD; dots represent individual rats. Exact *p* values are shown on the graph.

Serum levels of LDH (*p* = 0.001) and cTnI (*p* = 0.006) increased in HF‐Sed versus control. After 8‐week HIIT, serum levels of LDH (*p* = 0.019, Figure 4a) and cTnI (*p* = 0.043, Figure 4b) decreased in HF‐HIT versus HF‐Sed.

### 3.5 HIIT attenuates cardiac fibrosis

**FIGURE 5:**
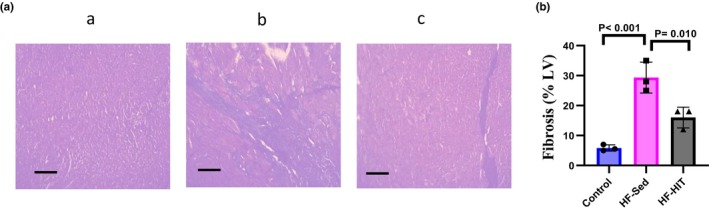
(a) 6‐μm transverse cardiac sections stained by Masson Trichrome (*n* = 4), the light blue area shows deposition of collagen fiber. Bar shows 100‐μm (×40). (A, control group, B, HF‐Sed group, and C, HF‐HIT group) (b) amount of fibrosis in all study groups. Data are expressed as mean ± SD.

Histological analysis showed that the interstitial fibrosis in the myocardium increased in HF‐Sed versus control (*p* = 0.0005). Our results indicated that myocardial interstitial fibrosis decreased in HF‐HIT versus HF‐Sed (*p* = 0.009, Figure 5). Finally, HIIT could improve interstitial fibrosis in HF rats.

### 3.6 HIIT attenuate oxidative stress

**FIGURE 6:**
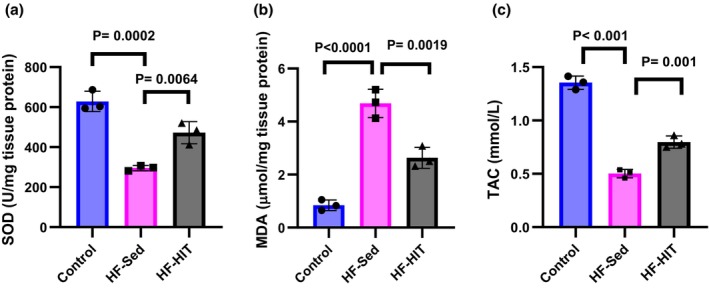
(a–c) Cardiac tissue levels of oxidative stress markers ((a) SOD, (b) MDA, and (c) TAC) (*n* = 4). Data are presented as means ± SD; dots represent individual rats. Exact *p* values are shown on the graph.

As Figure 6 indicates, heart tissue content of SOD decreased in HF‐Sed versus control (*p* = 0.0002), and increased in HF‐HIT versus HF‐Sed (*p* = 0.006, Figure 6a). Additionally, heart tissue content of MDA increased in HF‐Sed versus control and decreased in HF‐HIT versus HF‐Sed (*p* = 0.0019, Figure 6b).

### 3.7 HIIT attenuates iron deposition and suppresses ferroptosis by activating the SLC7A11/GPX4/NRF2 axis

**FIGURE 7:**
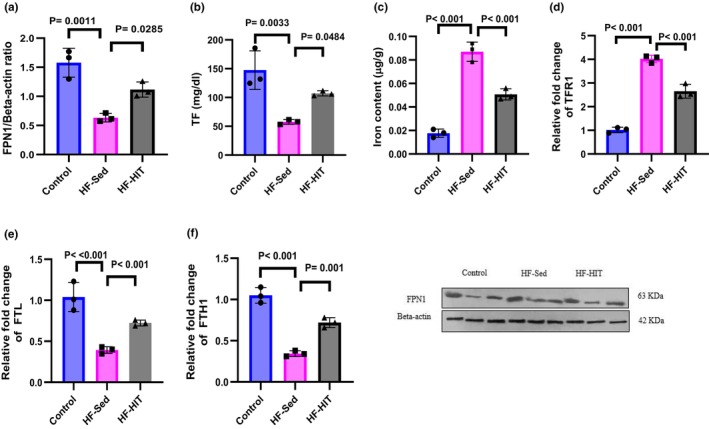
(a) Protein expression of FPN1. (b) Serum levels of TF. (c) Iron content. (d–f) TFR1 mRNA, FTL mRNA, and FTH mRNA (*n* = 3). Data are presented as means ± SD; dots represent individual rats. Exact *p* values are shown on the graph. The same β‐actin blot was used as a loading control for related target proteins processed on the same gel/membrane. Full‐length, uncropped Western blots with molecular weight markers are provided in Figure S1.

Protein expression of FPN1 decreased in HF‐Sed versus control (*p* = 0.001). While protein expression of FPN1 increased in HF‐HIT versus HF‐Sed (*p* = 0.028, Figure 7a). Serum levels of TF increased in HF‐HIT versus HF‐Sed (*p* = 0.048, Figure 7b). Iron content of heart tissues increased in HF‐Sed versus control (*p* = 0.0001). However, exercise intervention decreased iron content of heart tissue in HF‐HIT versus HF‐Sed (*p* = 0.001, Figure 7c). The TFR1 mRNA expression increased in HF‐Sed versus control (*p* < 0.0001), while FTL and FTH mRNA expression decreased in HF‐Sed versus control (*p* < 0.0001). Exercise intervention decreased mRNA expression of myocardial TRF1 in HF‐HIT versus HF‐Sed (*p* < 0.001, Figure 7d) while improving FTL and FTH mRNA expression in HF‐HIT versus HF‐Sed (Figure 7e,f).

**FIGURE 8:**
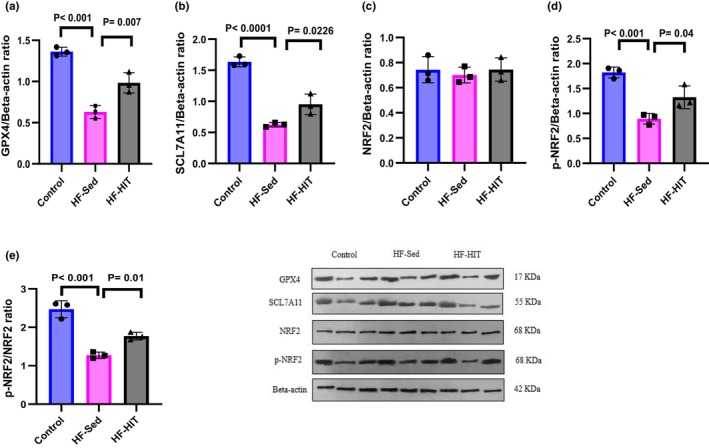
(a–e) Protein expression of GPX4, SCL7A11, NRF2, p‐NRF2, and p‐NRF2/NRF2 (*n* = 3). Data are presented as means ± SD; dots represent individual rats. Exact *p* values are shown on the graph. The same β‐actin blot was used as a loading control for related target proteins processed on the same gel/membrane. Full‐length, uncropped Western blots with molecular weight markers are provided in Figure S1.

Protein expression of GPX4, SCL7A11, p‐NRF2, and p‐NRF2/NRF2 ratio decreased in HF‐Sed versus control (*p* < 0.01). Our results show that 8‐week HIIT increased protein expression of GPX4, SCL7A11, p‐NRF2, and p‐NRF2/NRF2 ratio (*p* < 0.05, Figure 8a–e) in HF‐HIT versus HF‐Sed.

### 3.8 HIIT ameliorates ER stress by decreasing GRP78/PERK/ATF4 signaling pathway

**FIGURE 9:**
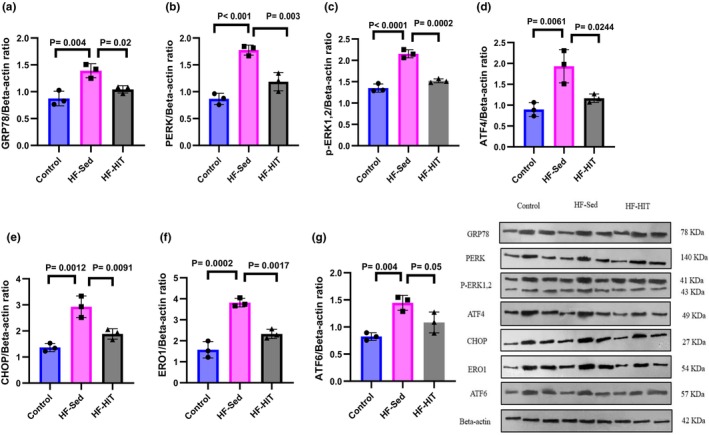
(a–g) Cardiac tissue levels of ER stress proteins ((a) GRP78, (b) PERK, (c) p‐ERK1/2, (d) ATF4, (e) CHOP, (f) ERO1, and (g) ATF6) (*n* = 3). Data are presented as mean ± SD; dots represent individual rats. Exact *p* values are shown on the graph. The same β‐actin blot was used as a loading control for related target proteins processed on the same gel/membrane. Full‐length, uncropped Western blots with molecular weight markers are provided in Figure S1.

Post‐HIT intervention attenuates unfolding protein response in myocardial tissue of HF rats. The results of Figure 9a–g indicated that protein expression of GRP78, PERK, p‐ERK1/2, ATF4, CHOP, ERO1, and ATF6 increased in HF‐Sed versus control (*p* < 0.01), while exercise training decreased protein expression of GRP78, PERK, p‐ERK1/2, ATF4, CHOP, ERO1, and ATF6 in HF‐HIT versus HF‐Sed (*p* < 0.05). Therefore, HIIT could ameliorate ER stress in HF rats.

## 4 DISCUSSION

**FIGURE 10:**
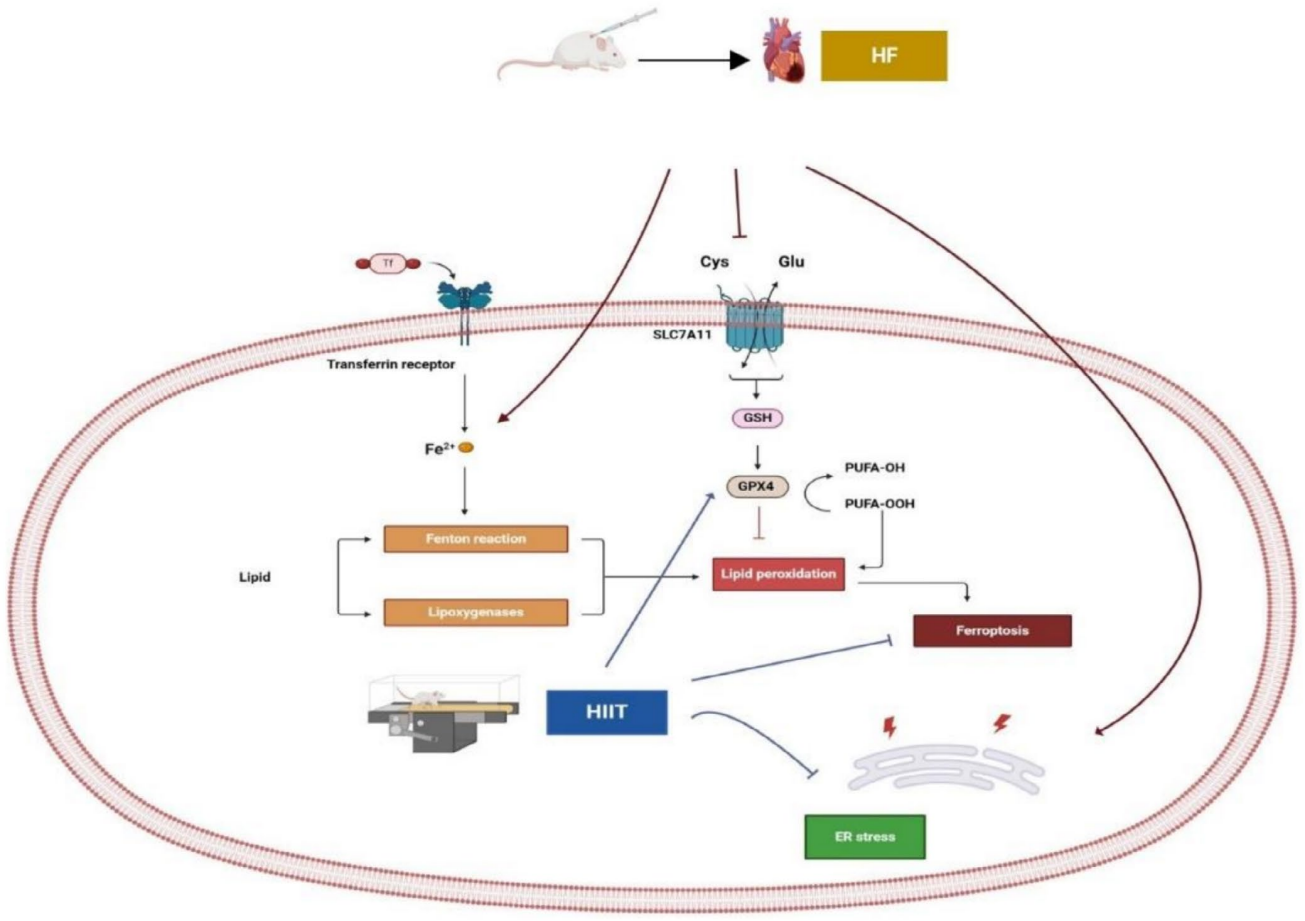
Schematic overview of the molecular pathways modulated by HIIT in HF. Created with BioRender.com.

The purpose of this study was to investigate the effect of HIIT on ferroptosis and ERS in myocardial tissue of rats with HF. The main findings of this study are as follows: (1) HIIT alleviates the iron overload, decreases the accumulation of lipid peroxidation, and improves antioxidant defense. (2) HIIT negatively regulated ferroptosis via an increase in the SCL7A11/GPX4/NRF2 pathway. (3) HIIT attenuates ERS with improved cardiac function in the animal HF model. Based on these results, increases of mRNA FTL, FTH (as iron‐storage protein ferritin), and protein expression of FPN1 in cardiomyocytes after HIIT may lead to decreased iron content in myocardial tissue. In this regard, the decrease of TFR1 mRNA in cardiomyocytes contributes to iron deposition (including attenuation of iron influx and promotion of iron efflux) after HIIT and is a reasonable cause of iron available reduction in cardiomyocytes. The reduction of TFR1 mRNA, which is identified as an importer of iron to cardiomyocytes, is associated with decreased ROS generation, leading to decreased susceptibility of cardiomyocytes to ferroptosis. To better visualize the interconnected pathways affected by HIIT in HF rats, Figure 10 provides an overview of the mechanisms we explored.

Iron is an essential micronutrient required in all mammalian cells for the proper function of many physiological processes, including antioxidant capacity, transport of oxygen, oxidative phosphorylation, and redox signaling (Lakhal‐Littleton, 2019). The accumulation of macrophages, fibrosis‐induced oxidative stress, excessive autophagy, and cardiomyopathy are important mechanisms of iron overload contributing to diastolic dysfunction in HF patients (Li et al., 2009). Previous studies provide strong evidence that the increase of TFR1 and the decrease of FPN1 in the HF are closely involved in the excessive ROS generation via the Fenton reaction, lipid peroxidation, and subsequently ferroptosis (Yang et al., 2022). TF is an essential glycoprotein responsible for transporting Fe^3+^ from the plasma to cardiomyocytes via TFR1. We speculated that the downregulation of TRF1 mRNA after HIIT may be a compensatory response against iron overload in the animal HF model. Additionally, the anti‐ferroptosis effects of HIIT induced by the increase of ferritin (FTL and FTH) and FPN eventually lead to decreased iron deposition in cardiomyocytes. Consistent with our results, a previous study indicated powerful evidence for the anti‐ferroptosis of moderate aerobic training in traumatic brain injury through invert iron overload by increasing the FTH and FPN1 and decreasing the levels of TFR1 (Chen et al., 2023).

The lipid peroxidation and inactivation of antioxidant defense are two key elements of the ferroptosis that increased in HF. Our results indicated that HIIT decreased lipid peroxidase biomarker and increased antioxidant defense after HF. As described above, the expression levels of intracellular enzyme GPX4, SLC7A11, p‐NRF2, and SOD increased in the heart tissue of rats with HF after HIIT. The cystine‐glutamate antiporter (System Xc^−^) is an important antioxidant system in cells, composed of SLC3A2 and SLC7A11, which mediates the exchange of glutamate and cystine across the plasma membrane (at a 1:1 ratio) to synthesize glutathione. The functional failure of system Xc^−^ increases the susceptibility of cardiomyocytes to ferroptosis due to decreased intracellular cystine and subsequent reduction of glutathione synthesis (Lee et al., 2021; Sun et al., 2020; Wang et al., 2019). The reduced expression of GPX4 in cardiomyocytes causes dysregulation of iron metabolism and accumulation of phospholipid hydroperoxides, leading to HF in rats (Bachmann et al., 2022). If the phospholipid hydroperoxides cannot be cleared rapidly by antioxidant molecules, the result is the propagation of lipid peroxidation and the formation of a toxic aldehyde such as MDA. Accumulation of lipid peroxidation products ultimately results in ferroptotic cell death by a decrease in membrane integrity and permeability (Jiang et al., 2021). Furthermore, NRF2 is an essential intercellular antioxidant defense by downstream target genes (Fan et al., 2017). Under oxidative stress, NRF2 separates from the Keap1‐NRF2 complex and translocates into the nucleus. In the nucleus, NRF2 binds to the antioxidant‐responsive element to promote various genes, including ferritin and heme oxygenase‐1 (HO‐1) (Sun et al., 2016). Additionally, NRF2 could directly bind to the antioxidant‐responsive element sequence of SLC7A11 and GPX4 subunit promoter to promote expression of SLC7A11 and GPX4 (Carpi‐Santos & Calaza, 2018; Dodson et al., 2019). The NRF2/SLC7A11/GPX4 pathway decreases in HF and HIIT may exert anti‐ferroptosis effects partly by a decrease in MDA and restoration of the impaired NRF2/SLC7A11/GPX4 pathway. Consistent with our results, evidence indicated that aerobic training decreased ferroptosis cell death via improved function of the NRF2/SLC7A11/GPX4 pathway in brain injury (Chen et al., 2023; Liu et al., 2022).

The regulation of ferroptosis can affect the level of oxidative stress in cardiomyocytes. As we know, the increase of ROS production is a pathophysiological characteristic in HF patients, which is facilitated by iron overload through the Fenton reaction (Zeng et al., 2019). Miyamoto et al. reported that increases of Fe^2+^ overload and upregulation of HO‐1 were observed in the ER of cardiomyocytes, triggering ferroptosis after ischemia/reperfusion (Miyamoto et al., 2022). ROS generation‐induced iron overload in cardiomyocytes or the ER can directly lead to oxidative damage of tissue, activating the UPR mechanism (Li, Li, et al., 2020). The UPR generally sense the misfolding protein in the ER through dissociates of GRP78 from three sensors and activates them (Hetz & Papa, 2018).

In the present study, we detected that the protein expression of GRP78/PERK/ATF4/CHOP/ERO1, ATF6, and p‐ERK1/2 increased in the heart of rats with HF. However, HIIT intervention mitigates ERS via decreased protein expression of GRP78/PERK/ATF4/CHOP/ERO1, ATF6, and p‐ERK1/2 in the heart of rats with HF. Finally, reduction of ferroptosis and ERS‐induced HIIT resulted in decreases in cardiac fibrosis and cardiac function.

Earlier research has indicated the positive effect of exercise training in possibly attenuating the ERS in heart disease and nonalcoholic fatty liver disease (Bozi et al., 2016; Cai et al., 2020; Ma et al., 2021; Yuan et al., 2022). Ma et al. reported that aerobic exercise (8‐week, 12–15 m/min, 1 h/day, 5 days/week) improved cardiac function via the decrease of p‐PERK and its downstream pathways eIF2/p‐eIF2/CHOP in rats with transverse aortic constriction (Ma et al., 2021). Cai et al. demonstrated that aerobic exercise training (6 weeks, 12 m/min, 1 h/day, 5 days/weeks) significantly improved cell death and cell viability in post‐myocardial infarction. Additionally, they found aerobic exercise training decreased GRP78 and CHOP through increased thioredoxin1 and thioredoxin interacting protein (Cai et al., 2020).

The link between ERS and ferroptosis is now described. In particular, Li et al. have shown that inhibiting the ATF4/CHOP pathway in rat models of diabetic myocardial infarction could not ameliorate the ferroptosis, while a ferroptosis inhibitor could reduce the occurrence of ERS, oxidative stress, and cardiomyocyte injury (Li, Li, et al., 2020). It was reported that inhibiting the system Xc^−^ not only increased ferroptosis but was also associated with activating the PERK/eIF2/CHOP pathway (Dixon et al., 2014). Additionally, it is suggested that ATF4 acts as a regulatory expression of SLC7A11 that demonstrates the feedback pathway between ferroptosis and ERS (Chen et al., 2017). The pharmaceutical agent of a ferroptosis inducer such as artesunate could activate a gene‐dependent ATF4/CHOP pathway such as HO‐1 and ERO1 (Lee et al., 2018). Activation of ERO1 induces the transcription factor CHOP, leading to augmented ERS. HIIT intervention reduces protein expression of ERO1 in heart tissue of rats with HF. One study indicated that HIIT reduces protein expression of the PERK/ATF4/CHOP signaling pathway in liver tissue of obese rats, and it suggests that high‐intensity training has a greater effect than moderate‐intensity training in upregulating SOD, catalase levels, and oxidative metabolism enzymes (Yuan et al., 2022). In addition, a study suggests that HIIT has a greater effect on CHOP, ATF4, ATF6, GRP78, p‐PERK/PERK, and p‐eIF2alpha/eIF2alpha in intermittent hypoxia than moderate‐intensity training (Bourdier et al., 2016). Consistent with previous results, we suggested that HIIT intervention attenuated ROS generation via increased SOD and reduction of MDA in cardiomyocytes of a rat model with HF. We speculated that ferroptosis was ameliorated by reduction of ERS and increase of antioxidant defense.

This study has several limitations. First, flow cytometry to determine ferroptotic cell and immunohistochemical staining with DAPI are often employed to evaluate GPX and nuclear translocation of NRF2. Insufficient financial resources prevented our use of flow cytometry and DAPI staining. Second, the beneficial effect of HIIT may involve multiple biological processes and multiple interaction mechanisms in rats with HF. However, we cannot exclude factors such as anti‐apoptosis, anti‐inflammation, and mitochondrial function as these might contribute to ferroptosis and need to be explored further. Despite our best efforts, we were unable to adequately address the sex dimensions in our research, which may have limited the generalizability of our findings. Since it could affect how the results are interpreted and applied, this constraint needs to be noted.

## 5 CONCLUSION

We report that 8 weeks of HIIT plays a key role in attenuating iron overload by increase of FPN1 and ferritin and reduction of TFR1. Our findings provide compelling evidence that the increase of GPX4, p‐NRF2, and SLC7A11 induced by HIIT decrease the heart's susceptibility of cardiomyocytes to ferroptosis. Additionally, the decrease of GRP78, ATF6, PERK, ATF4, CHOP, and ERO1 signaling pathway after HIIT reduces ERS.

## AUTHOR CONTRIBUTIONS

Aida Sabouri was involved in methodology, formal analysis, investigation, and writing—original draft preparation; Soheila Adeli was involved in formal analysis, investigation, funding acquisition, resources, and writing—review and editing; Abbasali Gaeini was involved in conceptualization, writing—review and editing, and supervision. Alireza Ghardashi Afousi was involved in conceptualization, methodology, writing—original draft preparation, writing—review and editing, resources, and supervision.

## FUNDING INFORMATION

This work was supported by Electrophysiology Research Center (Grant Number: 1402‐2‐233‐67589) Neuroscience Institute, Tehran University of Medical Science, Tehran, Iran.

## CONFLICT OF INTEREST STATEMENT

The authors declare no conflict of interest in this article.

## ETHICS STATEMENT

The animals used in this study were provided by the Research and Production Complex of Pasteur Institute of Iran. All animal experimental protocols were approved by the Ethics Committee of the Tehran University of Medical Sciences, Tehran, Iran (ethical approval number: IR.TUMS.AEC.1402.097). All animal experiments in this study followed the ARRIVE guidelines and complied with the National Research Council's Guide for the Care and Use of Laboratory Animals.

## Supporting information


Appendix S1.

## Data Availability

Data available on request from the authors.
