# Supplementary material for: High‐intensity interval training attenuates cardiac injury by targeting ferroptosis and endoplasmic reticulum‐stress in male rats with heart failure
Source: Physiol Rep. 2025 Sep 25;13(18):e70580. doi: 10.14814/phy2.70580 (PMC12463575; doi:10.14814/phy2.70580)
Supplement: Supplementary file 1 — Appendix S1. [file PHY2-13-e70580-s001.docx]

250 KDa


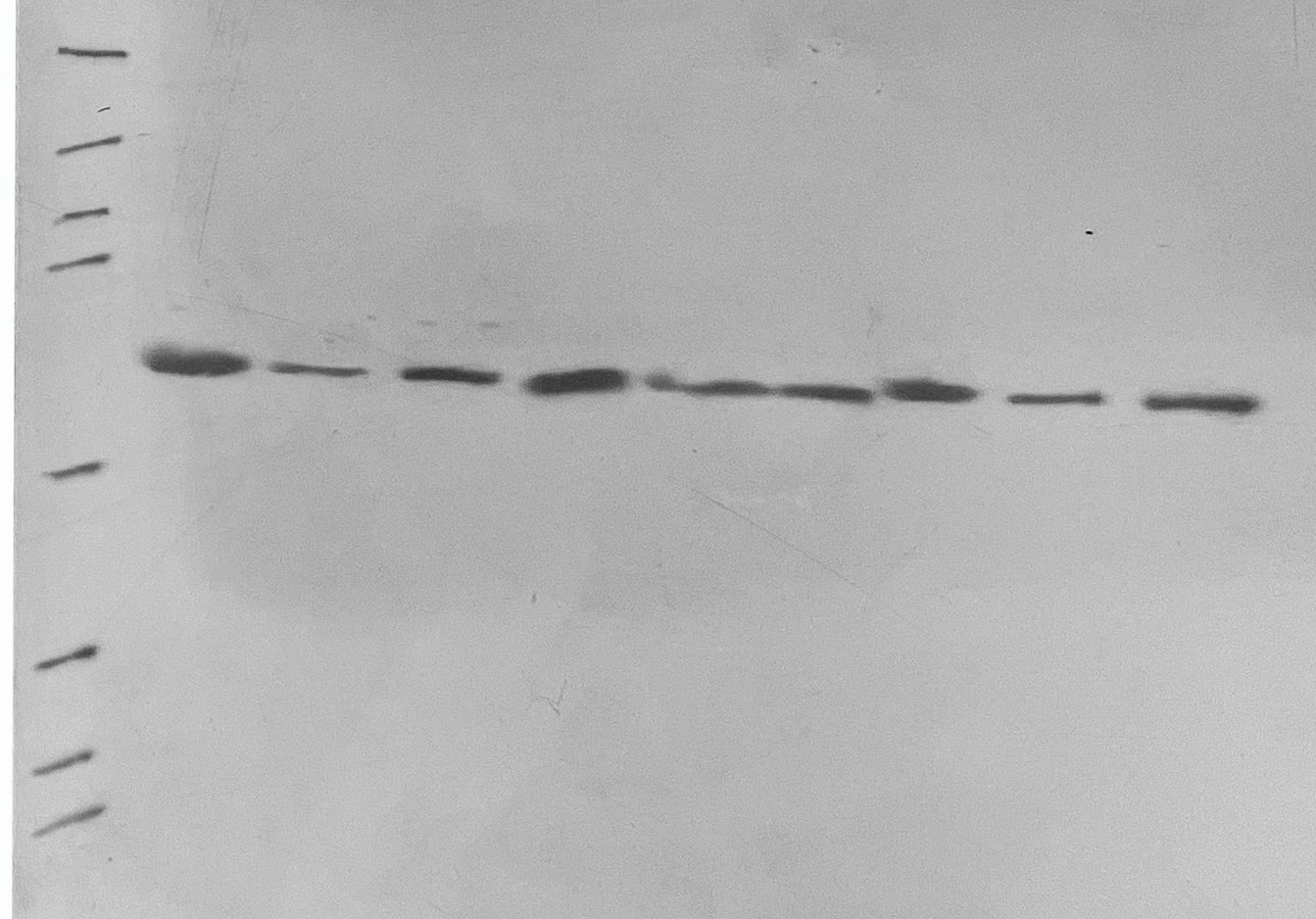


150 KDa

75 KDa

100 KDa

63 KDa

50 KDa

37 KDa

25 KDa

20 KDa

Fig. 7 A. FPN1


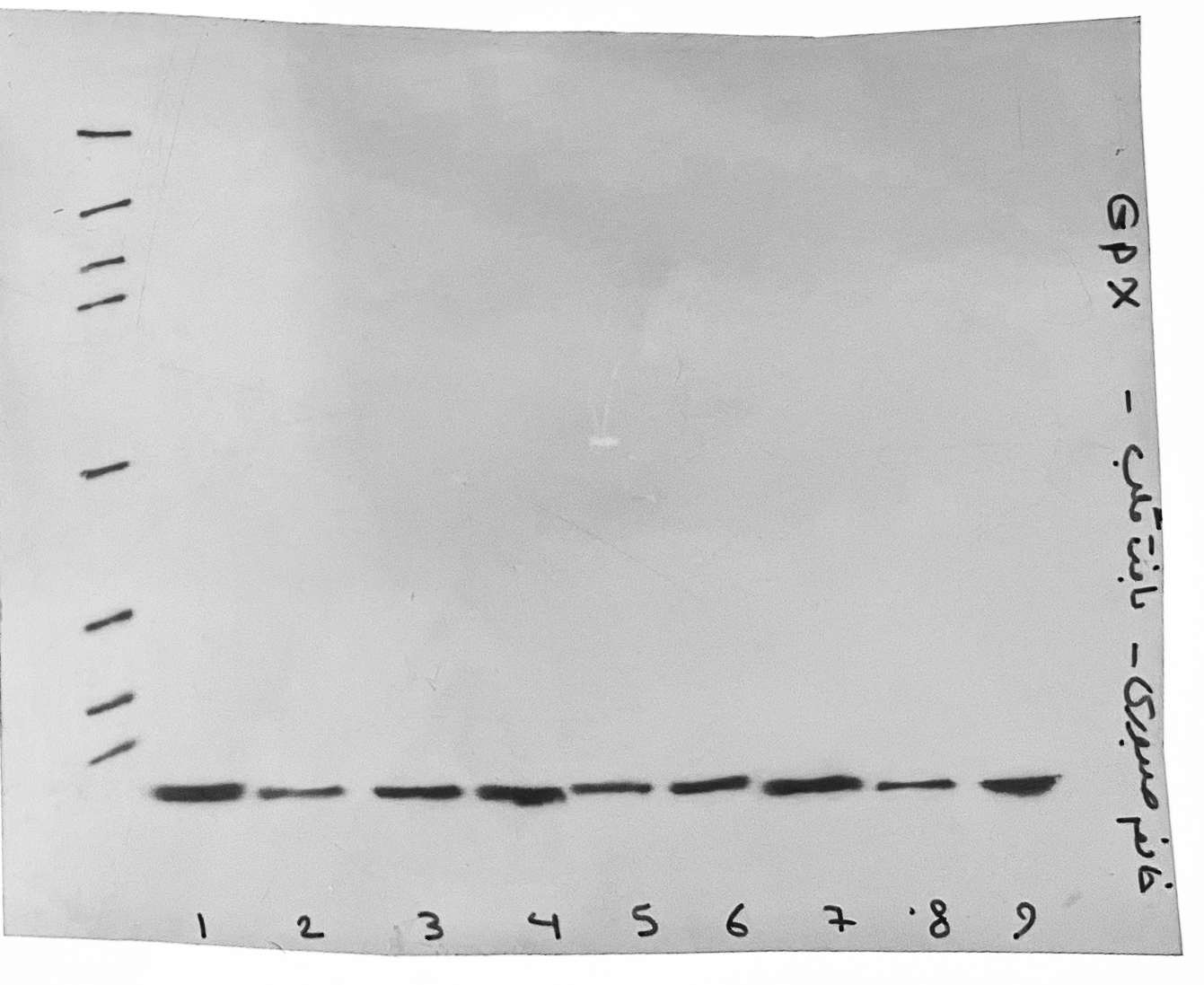


250 KDa

150 KDa

100 KDa

75 KDa

50 KDa

37 KDa

20 KDa

25 KDa

17 KDa

Fig.8 A. GPX4


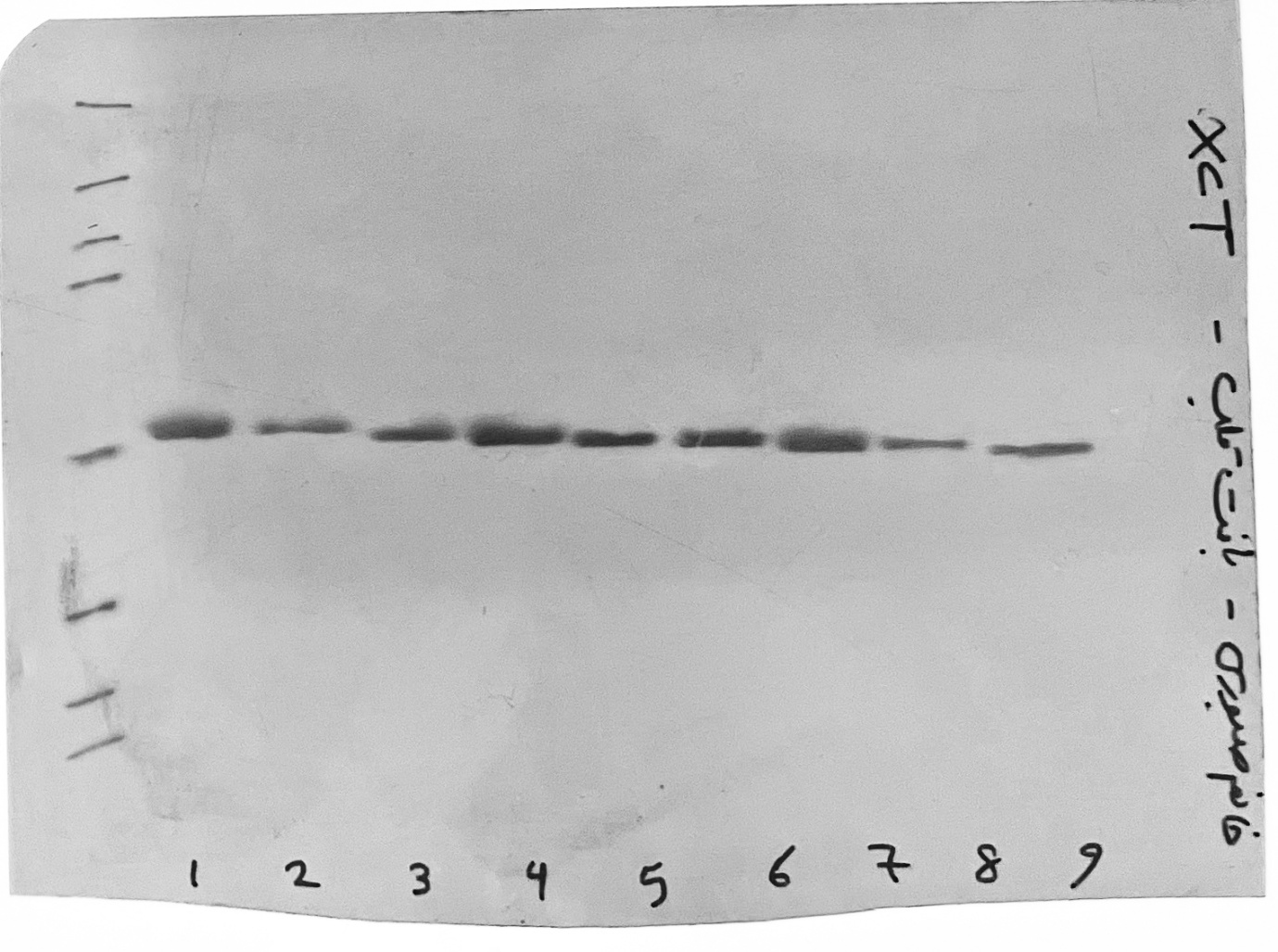


250 KDa

150 KDa

100 KDa

75 KDa

55 KDa

50 KDa

37 KDa

20 KDa

25 KDa

Fig.8 B. SLC7A11


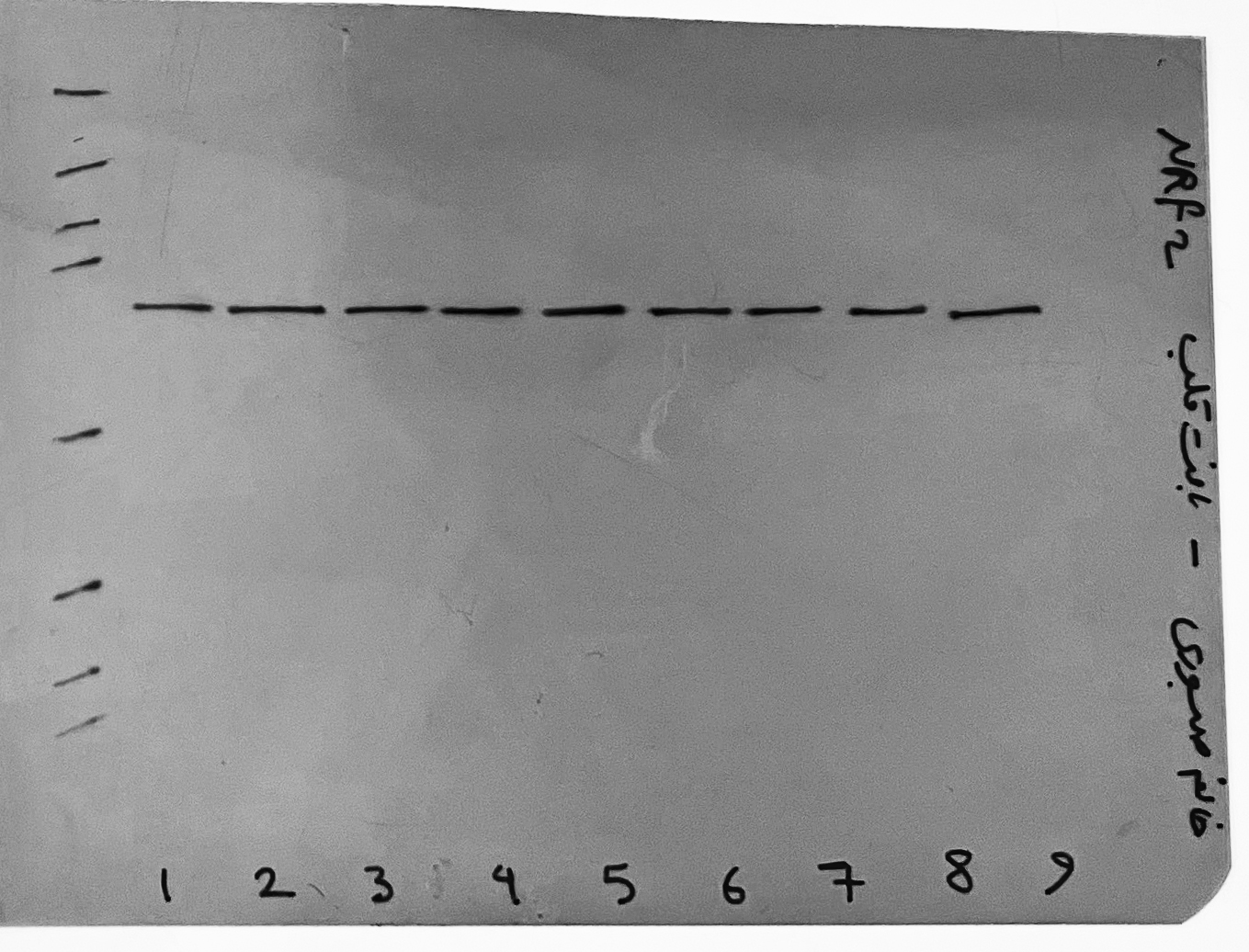


250 KDa

150 KDa

100 KDa

68 KDa

75 KDa

50 KDa

37 KDa

20 KDa

25 KDa

Fig.8 C. NRF2


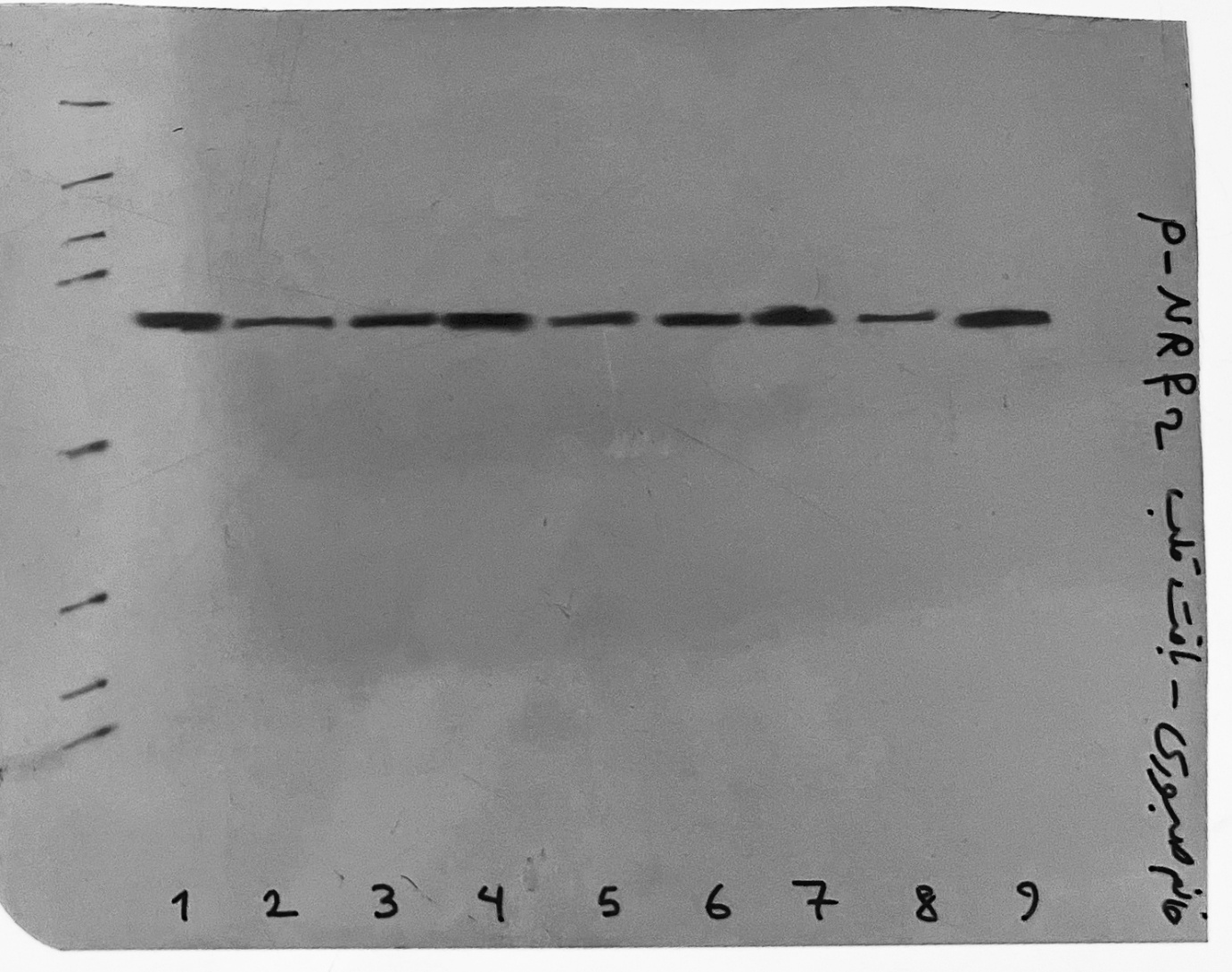


250 KDa

150 KDa

100 KDa

68 KDa

75 KDa

50 KDa

37 KDa

20 KDa

25 KDa

Fig.8 D. p-NRF2


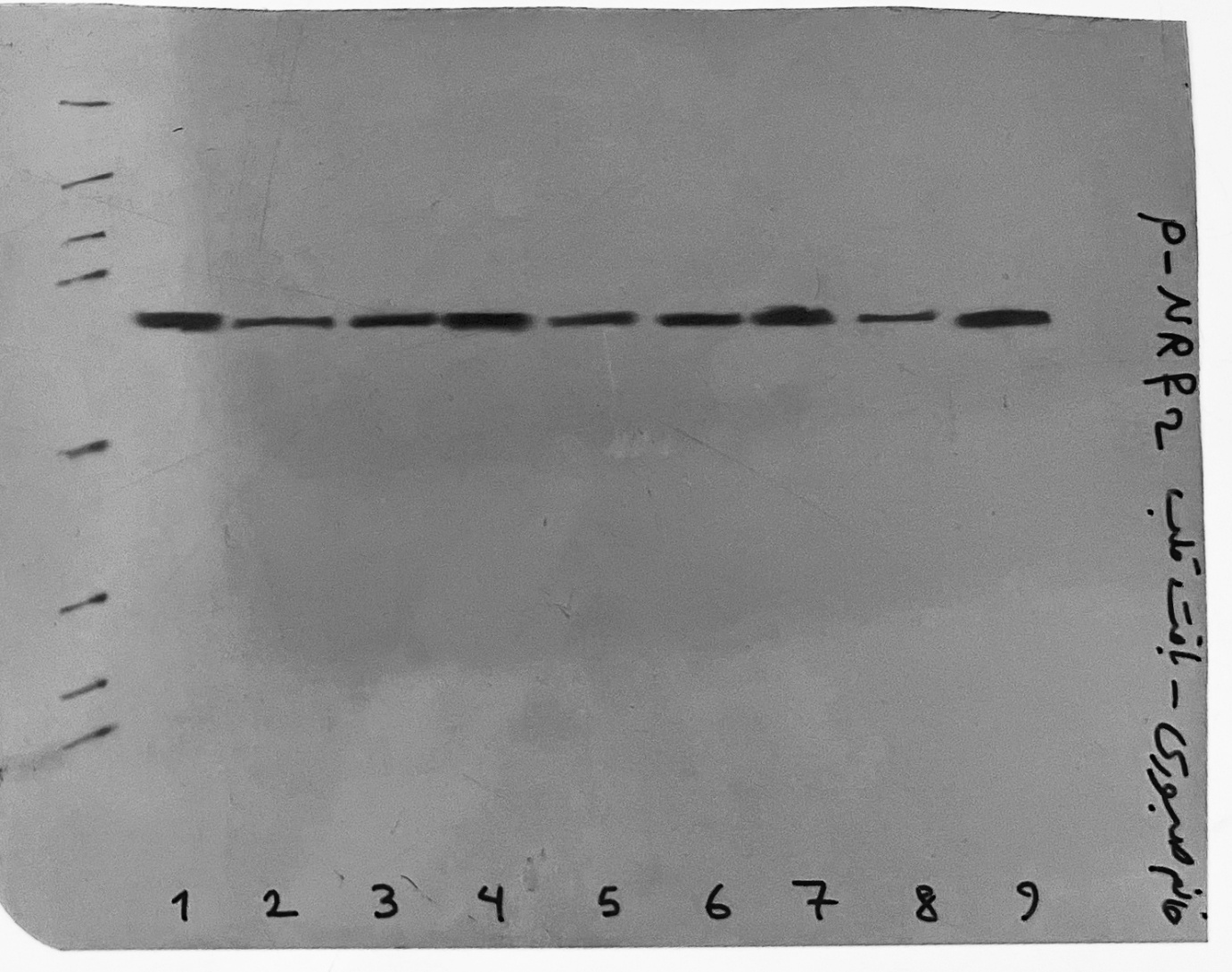


250 KDa

150 KDa

100 KDa

68 KDa

75 KDa

50 KDa

37 KDa

20 KDa

25 KDa

Fig.8 D. p-NRF2


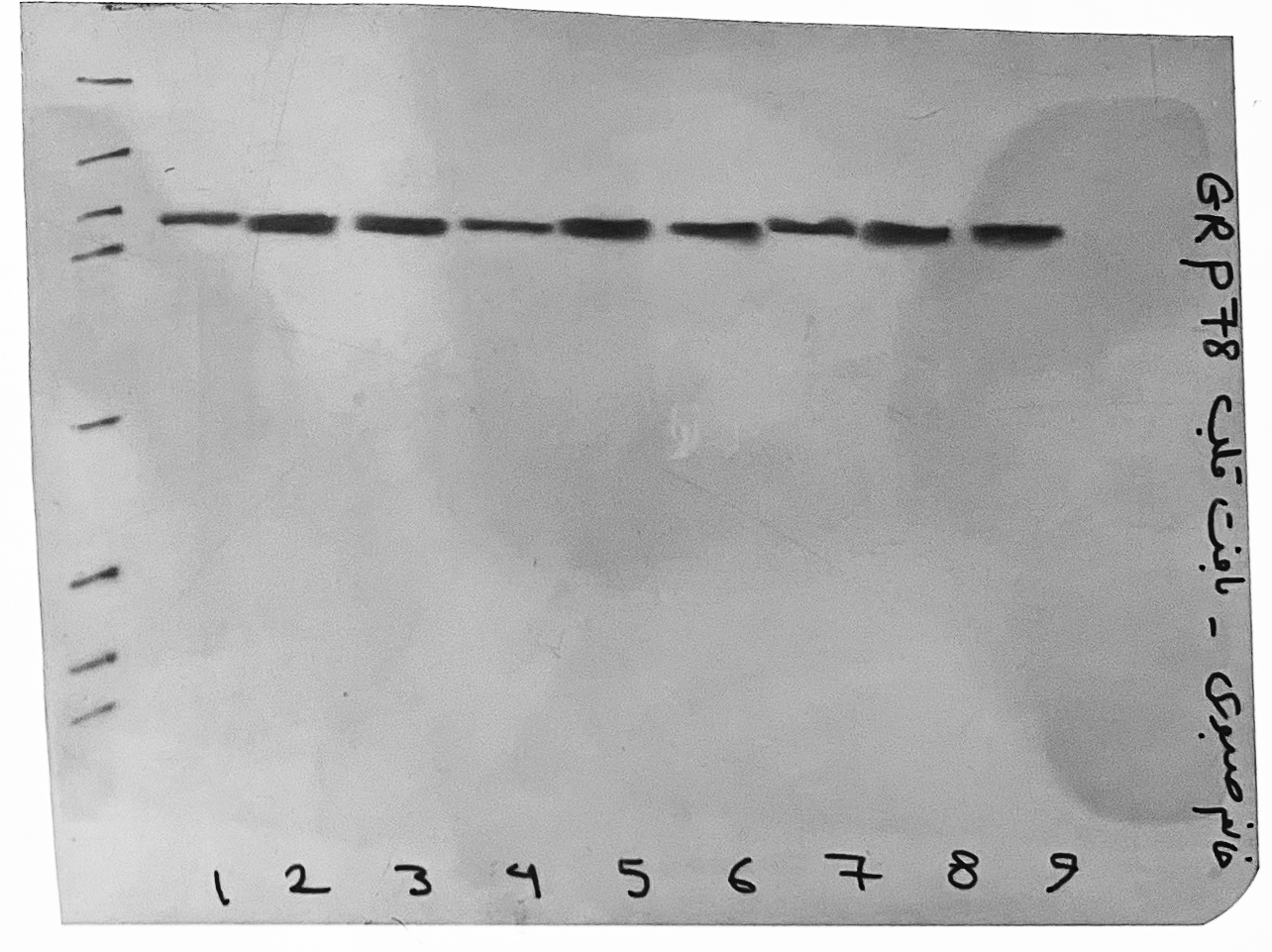


250 KDa

150 KDa

78 KDa

100 KDa

75 KDa

50 KDa

37 KDa

20 KDa

25 KDa

Fig.9 A. GRP78


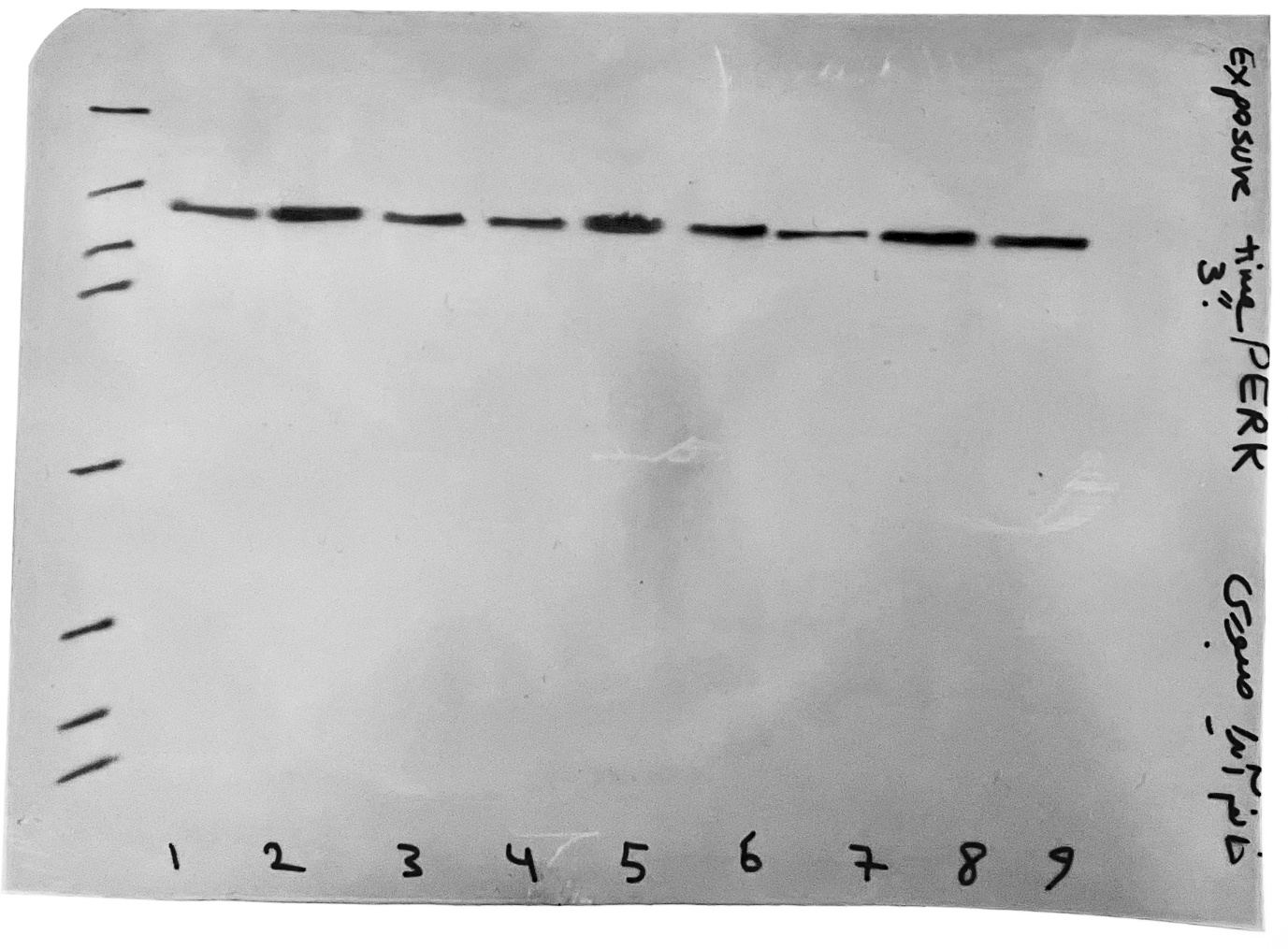


250 KDa

150 KDa

125 KDa

100 KDa

75 KDa

50 KDa

37 KDa

20 KDa

25 KDa

Fig.9 B. PERK


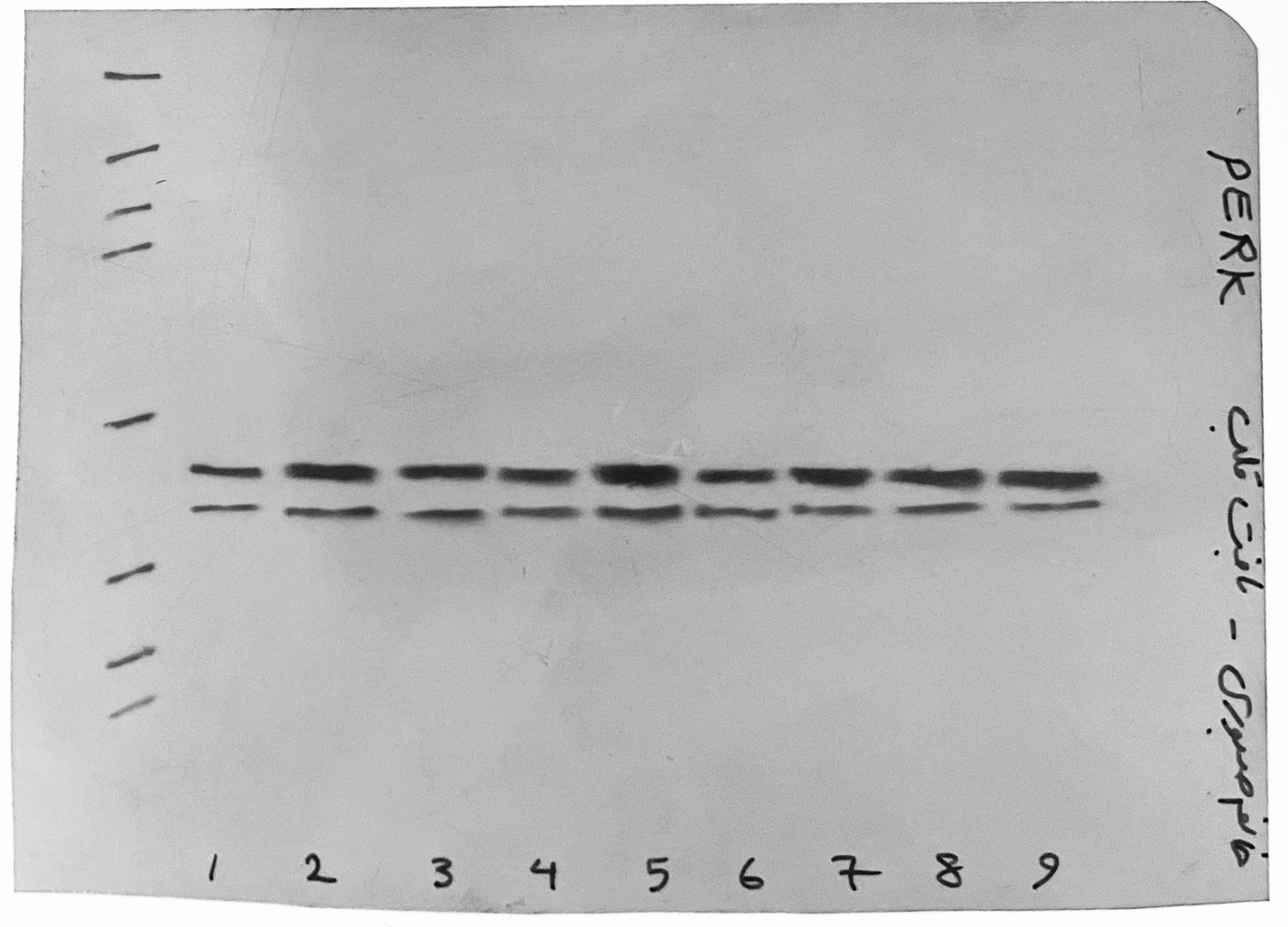


250 KDa

150 KDa

100 KDa

75 KDa

41 KDa

50 KDa

43 KDa

37 KDa

25 KDa

20 KDa

Fig.9 C. p-ERK1,2


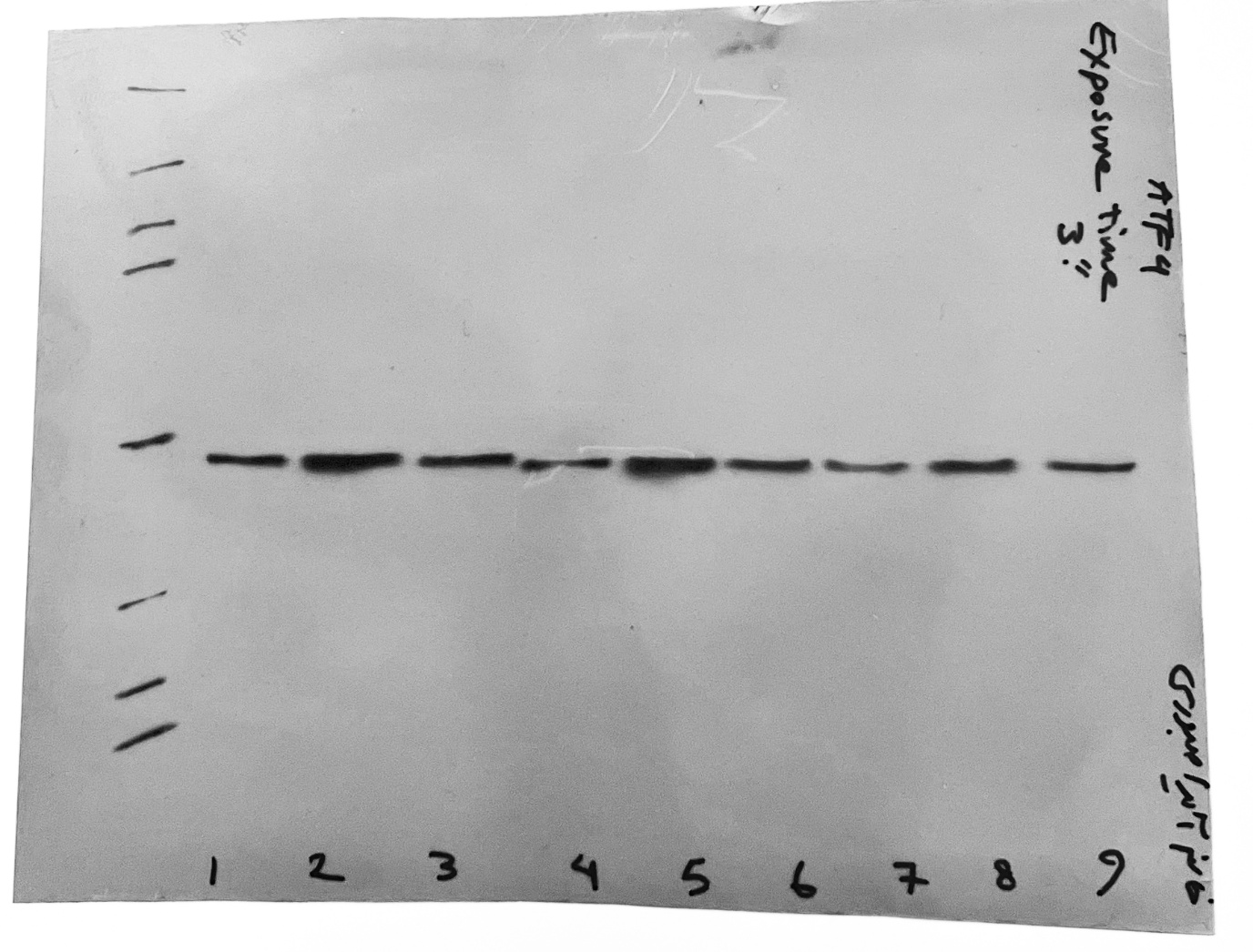


250 KDa

150 KDa

100 KDa

75 KDa

49 KDa

50 KDa

37 KDa

25 KDa

20 KDa

Fig.9 D. ATF4


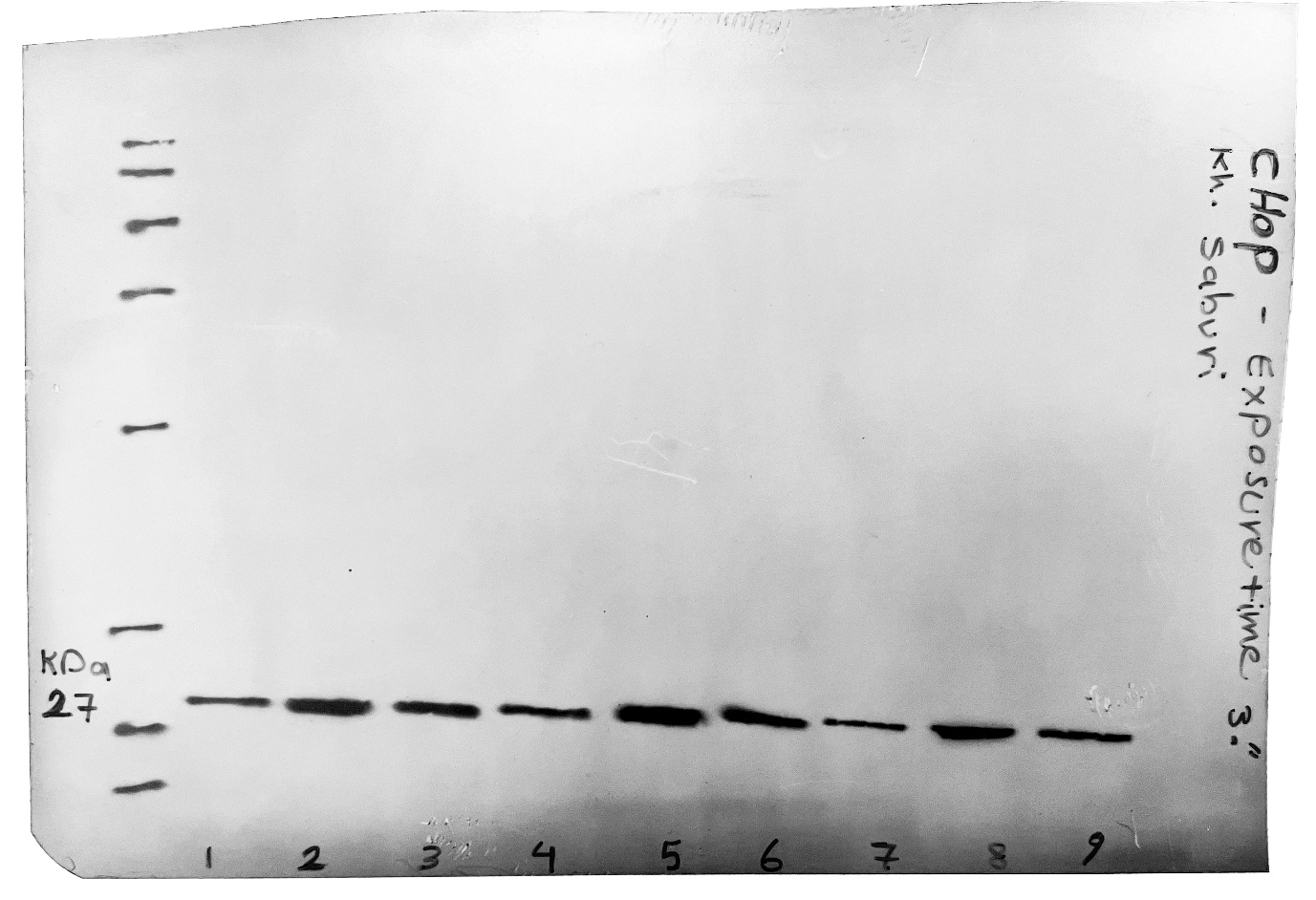


150 KDa

250 KDa

100 KDa

75 KDa

50 KDa

37 KDa

27 KDa

25 KDa

20 KDa

Fig.9 E. CHOP


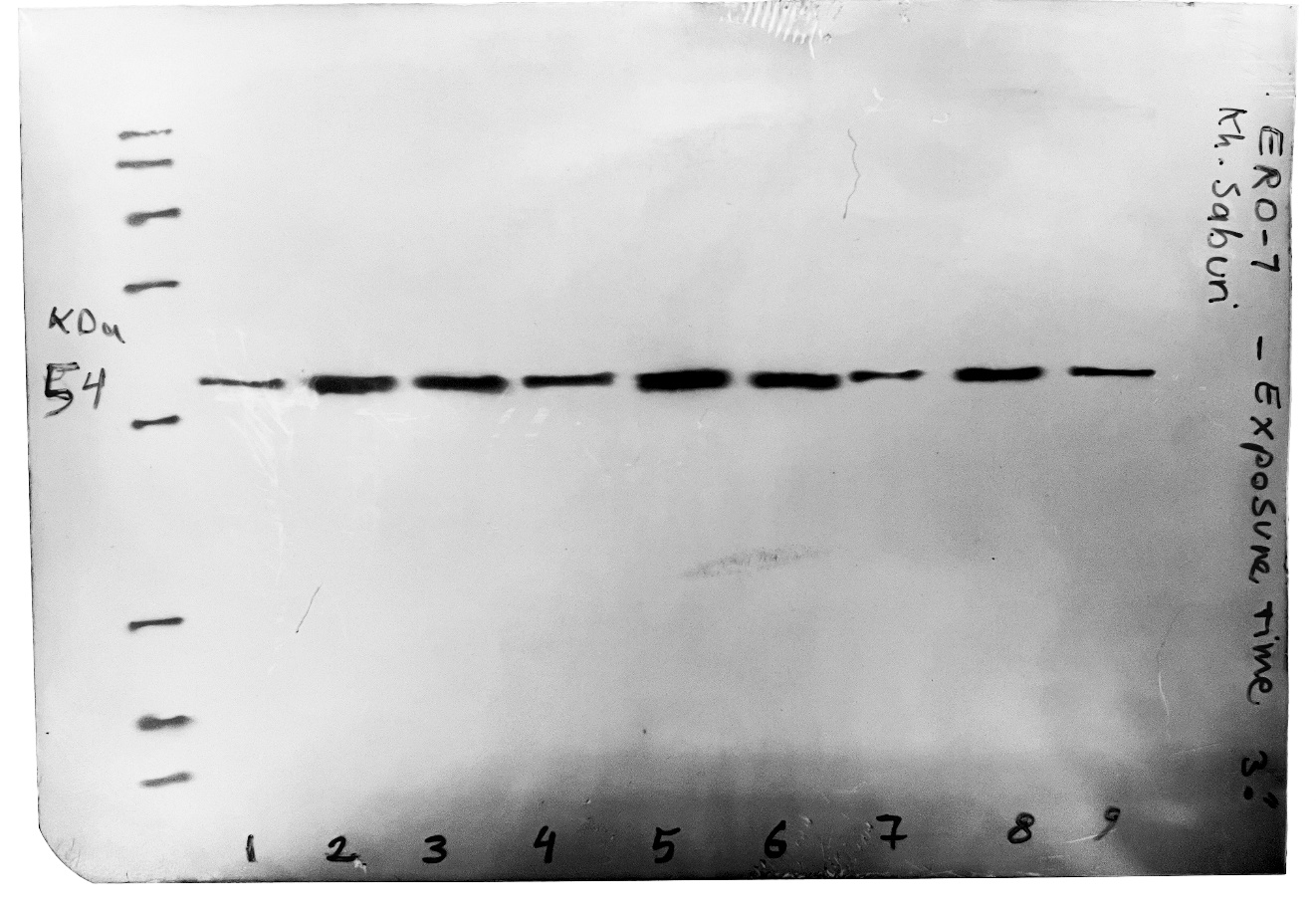


150 KDa

250 KDa

100 KDa

75 KDa

54 KDa

50 KDa

37 KDa

25 KDa

20 KDa

Fig.9 F. ERO1


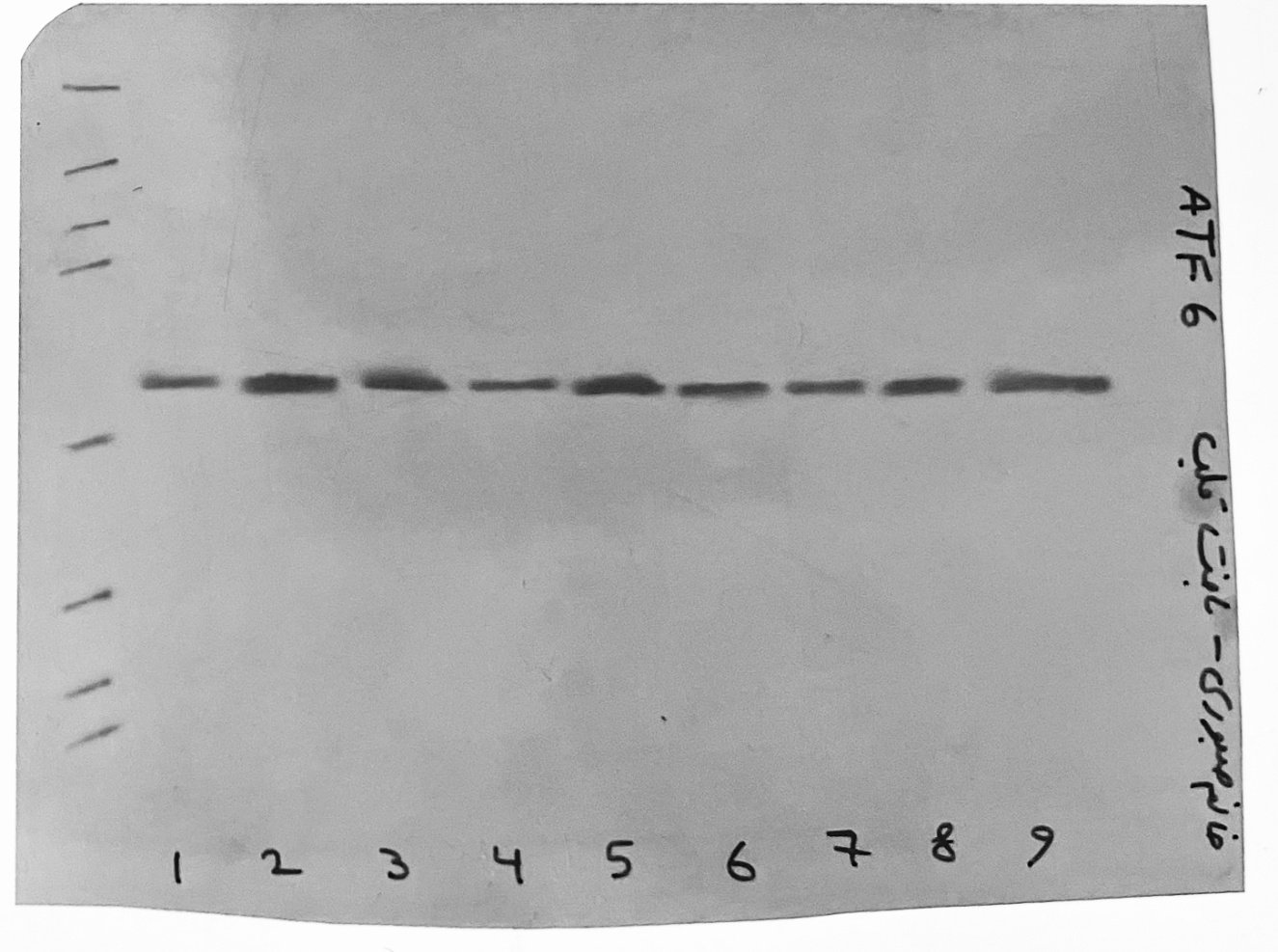


250 KDa

150 KDa

100 KDa

75 KDa

57 KDa

50 KDa

37 KDa

25 KDa

20 KDa

Fig.9 G. ATF6
